# A Theory of Planned Behaviour-Based Intervention on Dietary Supplement Use Among Varsity Athletes: A Pilot Randomized Controlled Trial

**DOI:** 10.3390/sports14080321

**Published:** 2026-08-01

**Authors:** Jana Daher, Jess Haines, Margo Mountjoy, Dalia El Khoury

**Affiliations:** 1 Department of Family Relations and Applied Nutrition, University of Guelph, Guelph, ON N1G 2W1, Canada; jdaher@uoguelph.ca (J.D.); jhaines@uoguelph.ca (J.H.); 2 Department of Family Medicine, McMaster University, Hamilton, ON L8P 1H6, Canada; mmsport@mcmaster.ca

**Keywords:** Theory of Planned Behaviour, dietary supplements, varsity athletes, intention-behaviour gap, nutrition education, behavioral control, consumption

## Abstract

Background/Objectives: The rising prevalence of dietary supplement use among athletes raises concerns, given the risks associated with unnecessary or excessive use. This study evaluated the impact of a Theory of Planned Behaviour (TPB)-based online educational intervention, “Nutrition for Athletes”, on dietary supplement use among varsity athletes at a Canadian university. Methods: Thirty varsity athletes were randomized into intervention (n = 18) and control (n = 12) groups. The intervention was delivered online over four weeks and consisted of four units that covered topics related to sports nutrition, hydration in sports, dietary supplements, and risks associated with dietary supplement use. Dietary supplement use was measured at baseline and post-intervention using self-reported questionnaires. Results: The most commonly used supplements at baseline were vitamin D (57%), vitamin C (53%), sports drinks (50%), protein bars, powders, and shakes (43%), and whey protein (40%). Binary logistic regression revealed a reduced likelihood of dietary supplement use in the experimental group compared to the control group post intervention, but this difference was not statistically significant (OR = 0.18, 95% CI = 0.01 to 1.76, *p* = 0.14). There was also no significant change in the behaviour of dietary supplement use within the experimental group, when comparing baseline to post-intervention (OR = 0.12, 95% CI = 0.01 to 1.11, *p* = 0.06). Conclusions: These findings reflect the potential existence of an intention-behaviour gap. Future interventions may benefit from incorporating strategies that address control-related barriers.

## 1. Introduction

Dietary supplementation is generally needed for people following a low-energy diet, eliminating at least one food group from their diets, using severe weight-loss practices, or consuming a high-carbohydrate diet poor in vitamins and minerals [1]. Nevertheless, the utilization of dietary supplements extends beyond these specific groups, encompassing a broader demographic. This broader usage can be attributed to a surge in the popularity of health and wellness trends, coupled with a collective desire to enhance overall well-being or athletic performance. However, the spectrum of what can be considered a dietary supplement is wide and differs from one country to another [2]. The broad nature of what qualifies as a dietary supplement, along with the lack of clear categorization, make it challenging to design research and compare research findings effectively [3].

The surge in dietary supplement use is particularly notable in the athletic population, who are more likely to consume sports supplements to improve performance, enhance recovery, and improve overall health [4]. While the efficacy of sports supplements is not always backed by scientific evidence, and their safety is questioned both in terms of potential health risks and the risk of unintentional doping violations, the prevalence of dietary supplement use among athletes continues to rise [5].

Dietary supplements may be appropriate when medically or nutritionally indicated; however, unnecessary or unguided use may expose athletes to potential health risks and inadvertent doping. Many athletes use sports supplements without a clear need for them, often influenced by their peers [6]. Moreover, several studies have found that athletes’ information on dietary supplements usually comes from unreliable sources, such as their teammates, coaches, the internet, and social media [7]. This lack of credible guidance is concerning given that dietary supplement contamination with harmful substances prohibited by the World Anti-Doping Association (WADA), such as anabolic androgenic steroids, central nervous system stimulants, and diuretics, has been documented for more than two decades [8,9,10]. One study investigating sports supplements reported contamination rates as high as 58% [10]. Contamination can occur either due to inadequate manufacturing procedures or can be intentional by manufacturers to increase the effectiveness of supplements [11]. The high prevalence of dietary supplement consumption among athletes, especially those marketed to enhance performance, combined with a lack of industry regulations, puts them at a risk of inadvertent doping [9,12]. Inadvertent doping is the unintentional use of a prohibited substance. Current data have reported that consumers are unaware of the harmful potential of dietary supplements [13,14] and have unintentionally consumed supplements contaminated with anabolic steroids, prohormones, selective androgen receptor modulators (SARMs) and aromatase inhibitors that were not mentioned on the label [10,11,15]. A meta-analysis examining the prevalence of contamination in dietary supplements found that almost 28% of the examined supplements posed a potential risk of inadvertent doping [8]. They also found that 875 out of 3132 examined dietary supplements contained undeclared substances [8]. It is vital that athletes understand the risk of consuming dietary supplements, as trace amounts of prohibited substances can still result in a failed drug test even in quantities that are too low to have any actual physiological effects [16].

Despite this widespread use, many dietary supplements lack consistent scientific evidence supporting their effectiveness in enhancing athletic performance, recovery, or health outcomes [16]. It is critical to understand that most of the claims made about dietary supplements are unproven, and that there are no defined standards for the quality of the evidence required to support such claims [17,18]. For example, studies with numerous design and experimental flaws may still be presented as having sufficient scientific evidence to back a claim made by a dietary supplement company [18]. Therefore, it is not uncommon for scientific evidence purporting the usefulness of dietary supplements to emerge from the least rigorous studies [16]. A recent systematic review on the effectiveness of creatine supplementation in enhancing performance in active females, a supplement widely used by athletes, found that most studies did not show an improvement in performance compared to placebo [19].

In contrast to pharmaceutical products, which are subject to strict pharmaceutical rules based on premarketing authorization and end-product supervision, dietary supplement manufacturers and distributors are also not required to obtain Food and Drug Administration approval before selling them. Countries regulate dietary supplements on a national level, and regulations vary from one nation to another [3]. Evidently, dietary supplement regulation is lax; there are no global quality control rules, which explains why there are several substandard products throughout the world. While an ideal scenario would entail a comprehensive pre-market approval process where both products and manufacturing facilities undergo evaluation before dietary supplements are introduced to the market, practicality often prevents its widespread implementation [20].

Athletes may still invest in supplements that offer little to no measurable benefit, usually influenced by body image pressures related to muscularity. Collectively, these issues add another layer of complexity to the decision-making process and necessitate a need for interventions aimed at improving knowledge and promoting more informed, evidence-based decisions regarding dietary supplement use. Therefore, it is essential that these interventions be based on well-established theoretical frameworks that guide behaviour-change efforts.

While the Theory of Planned Behaviour is not a theory of behaviour change, it was proven to effectively serve as one of the most successful frameworks for behaviour change interventions [21]. Over the last three decades, extensive research using this theory has sparked across different applied fields, especially in terms of developing interventions [21,22,23,24,25]. Extended from the Theory of Reasoned Action, this theory proposes that human behavior is driven by intentions, which are influenced by attitudes, subjective norms, and perceived behavioral control [26]. Attitudes are a person’s evaluation of the likely consequences resulting from performing a behaviour. Subjective norms are the perceived social pressure to engage in or refrain from a certain behaviour. Perceived behavioral control is the perceived capability to perform a behaviour [27].

Ajzen, a social psychologist who developed the Theory of Planned Behaviour, differentiated between perceived behavioral control and actual behavioral control. Perceived behavioral control strengthens or weakens the relationship between attitude and subjective norm on intention, while actual behavioral control is assumed to influence the extent to which intention translates into behavior. Since we lack sufficient information about actual behavioral control (all factors that contribute to facilitating or inhibiting a certain behaviour), perceived behavioral control may be used as a proxy to help predict behaviour, assuming that actual behavioral control is well reflected by perceived behavioral control [28]. Even when individuals hold beliefs that may be inaccurate or biased, these beliefs will still shape their attitudes, perceived social pressure, and sense of control over their actions. Consequently, these beliefs form a behavioral intention that will drive their actions accordingly [26].

There have been several criticisms regarding the predictive ability of the Theory of Planned Behaviour [29]. The intention–behaviour correlation was reported to vary considerably among studies. Another frequent criticism of the theory is that it is too “rational”, and does not take into account human biases, such as motives, fear, anger, and other emotions that control decision making. However, Ajzen argues that those emotions typically operate in the background by influencing beliefs, which in turn influence intentions towards a given behaviour. Several interventions based on the Theory of Planned Behaviour were not successful in changing behaviour; however, theory advocates argue that this may be due to (1) loose application of the theory during intervention development and execution, (2) external barriers to behaviour change that affect actual behavioral control, and (3) the use of a wrong intervention type (e.g., focusing on modifying attitudes in subjects who already have favourable intentions but are unable to act due to control issues) [26].

Several interventions in the nutrition field have been guided by the Theory of Planned Behaviour. Jalilian et al. (2011) implemented this theory to design and test an educational intervention on the use of anabolic androgenic steroids among gym users [30]. While the education program did not improve subjective norms and perceived behavioral control of participants, it was successful in increasing their behavioral intentions to avoid using them. A Theory of Planned Behaviour-based online intervention that was developed to modify dietary supplement behaviour among high school athletes resulted in more athletes reporting future third-party tested supplement use [31]. Another study by Alami et al. (2019) examined the effectiveness of a Theory of Planned Behaviour-based educational intervention aimed at improving adolescent girls’ intentions to consume iron and vitamin D supplements in Iran. The intervention led to significant improvements in knowledge, subjective norms, perceived behavioral control, and behavioral intentions compared to the control group, with all constructs except attitude showing statistically significant changes [32].

Although previous TPB-based interventions have demonstrated improvements in knowledge, intentions, and related behavioural determinants, evidence regarding whether these changes translate into actual changes in dietary supplement use among athletes remains limited. Examining behavioural outcomes is important given that changes in intentions do not necessarily result in corresponding changes in behaviour. Therefore, this study aimed at evaluating the impact of the “Nutrition for Athletes” program, a Theory of Planned Behaviour-based pilot intervention that was successful in modifying varsity athletes’ intentions towards using dietary supplements, on dietary supplement use among the same population.

## 2. Materials and Methods

### 2.1. Participants and Study Design

**Figure 1:**
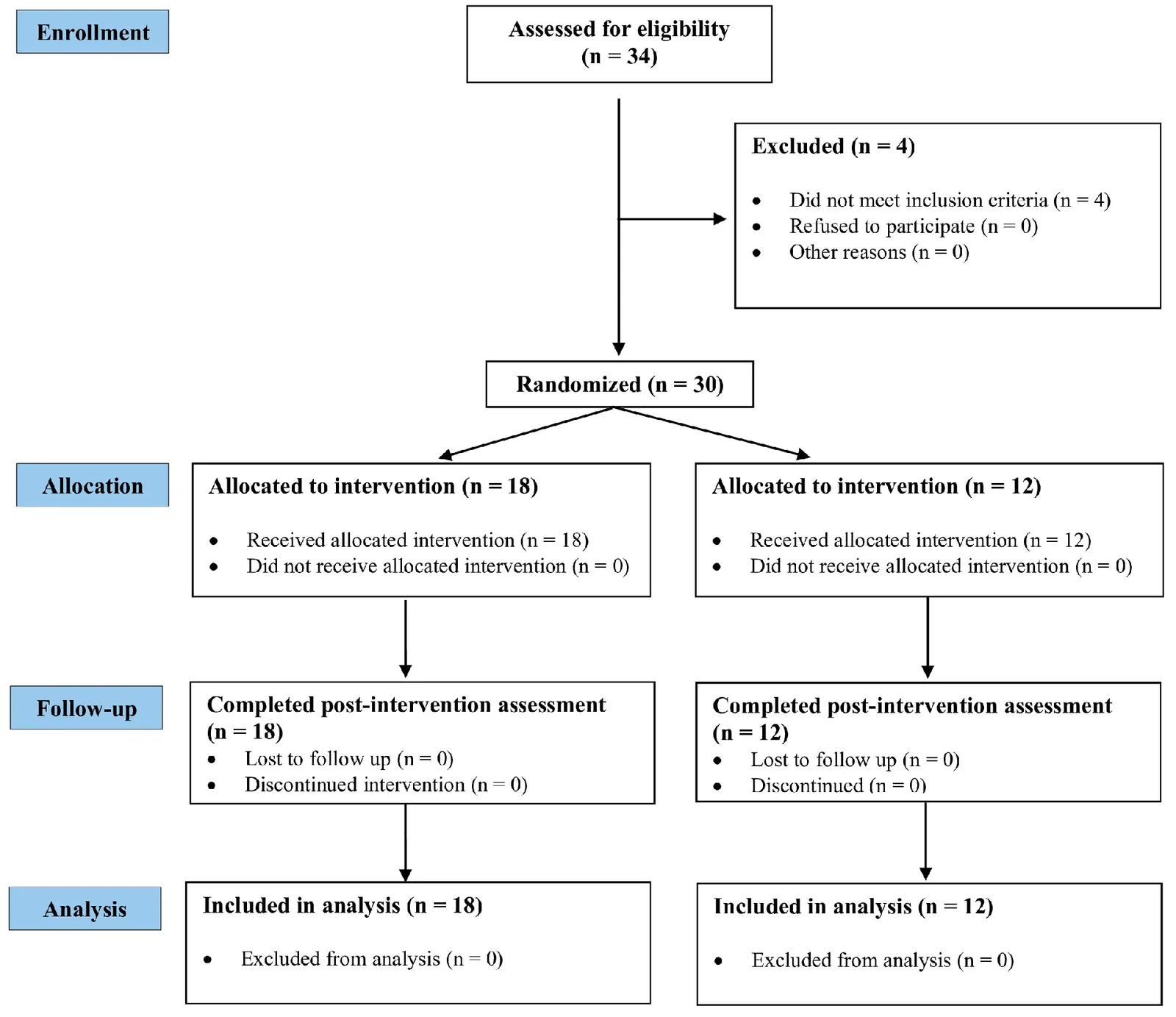
Flow of participants through the study.

This pilot study was carried out at the University of Guelph in Canada and registered on ClinicalTrials.gov (NCT05514808). Data was collected from 30 varsity athletes who were randomized through an online clinical trial randomization tool into an intervention group (n = 18) and a control group (n = 12) (Figure 1). Participants were enrolled prior to group allocation. Participating athletes were recruited through posters placed in common student spaces, email invitations sent via coaches and team staff, and social media posts shared on varsity teams’ social media accounts. To be eligible, participants had to be registered students at the University of Guelph, members of a varsity team, and fluent in English. All participants provided electronic informed consent and received a $50 Amazon gift card after completing the study. This study was approved by the University of Guelph Research Ethics Board (REB# 22-03-003). For more details, please see Daher et al. (2024) and Daher et al. (2025) [33,34].

### 2.2. Intervention Program and Assessment

The intervention consisted of a self-administered, online nutrition education program grounded in the Theory of Planned Behavior. The program was delivered via Courselink, the University of Guelph’s learning management system, and comprised four sequential units covering topics related to sports nutrition and dietary supplements. The first unit covered topics related to sports nutrition, energy needs, and timing of meals. The second unit highlighted the importance of hydration, ways to identify dehydration, and strategies to prevent it. The third unit introduced the definition of dietary supplements, provided examples, explained their use, and outlined their different categories. Risks associated with dietary supplement use were the focus of the fourth unit, covering topics such as doping, contamination of dietary supplements, fair play in sports, and the potential harms of unguided dietary supplement use.

Participants were required to complete each unit in sequence to gain access to the subsequent one. The program was designed to be completed over a four-week period. The reading time for each unit was 15–20 min. Each unit also included fun facts, myth busters, and quizzes. Progress of participants in the experimental group was monitored on CourseLink before administering the post-program questionnaire through ensuring that participants completed all modules. Those who had not completed all four units received email reminders before the questionnaire was distributed. Participants who had not completed the assigned modules were sent reminders. Six of the 18 participants in the experimental group required at least one reminder, with one participant requiring two reminders. For ethical considerations, participants in the control group were provided access to the intervention following the completion of the second questionnaire [33,34].

To evaluate dietary supplement use, an online questionnaire (supplement use questionnaire) was administered to all participants at baseline and again at four weeks. The questionnaire has been tested for validity and reliability on the target population [35]. Content validity was examined through expert evaluation, which assessed 12 factors, including relevance, brevity, repetition, order, response options, and bias. It included questions on participants’ demographics such as age, gender, medical conditions, parents’ or guardians’ education levels, ethnic background, smoking habits, and alcohol consumption. The questionnaire also included the types and frequency of use of dietary supplements, as well as the reasons for use and sources of information. The demographics section gathered information on participants’ age, gender, health status, parental or guardian education levels, ethnicity, smoking behaviors, and alcohol use.

### 2.3. Statistical Analysis

Statistical analyses were conducted using the statistical software package for social sciences SPSS (v.24 for MS Windows). Descriptive statistical analysis was used to determine the prevalence of dietary supplement use at baseline. There was one binary outcome (dietary supplement use), assessed at baseline and post-intervention; therefore, a binary logistic regression was performed to examine dietary supplement use differences between and within groups. Logistic regression models were adjusted for age, gender, medical condition, level of education of parents or guardians, ethnic background, smoking habits, and alcohol consumption. A 95% confidence interval was used to determine significance (*p* ≤ 0.05).

## 3. Results

### 3.1. Participant Demographics

A total of 30 participants completed the study. The mean age of participants was 20.6 years (SD = 2.9), with ages ranging from 18 to 30 years. The sample was predominantly female (70%). In terms of ethnicity, 67% identified as Caucasian, followed by 10% Southeast Asian, 10% West Asian, 3.4% Indigenous, 3.3% Latin American, and 3.3% Arab. An additional 3% preferred not to disclose their ethnicity. All participants reported non-smoking status, while 53% reported alcohol consumption. Parental or guardian education levels were reported as follows: a bachelor’s degree (43%), a graduate or professional degree (37%), a college diploma or certificate (17%), and a high school diploma or equivalent (3%).

### 3.2. Baseline Characteristics of Dietary Supplement Use

The consumption of different dietary supplement categories and types was assessed at baseline for all participants (Table 1). In total, 80% of participants reported using vitamin/mineral supplements. The most commonly used vitamins and minerals were vitamin D (57%) and vitamin C (53%). The main reasons reported for using vitamin/mineral supplements included maintaining good health, preventing nutrient deficiencies, supporting immune function, and enhancing energy levels.

Half of the participants have reported using protein supplements, mainly protein bars, powders, shakes (43%) and whey protein (40%). The main reasons reported for using protein supplements were to supply convenient forms of energy and/or macronutrients, increase or maintain muscle mass, maintain good health and support intense training regimens. A total of 60% of participants used carbohydrate supplements, mainly as sports drinks (50%). The main reasons reported for using carbohydrate supplements included increasing energy, supporting intense training regimens, promoting recovery, and enhancing competitive performances. Table 1 also depicts the prevalence of use of several other supplement types such as fatty acid supplements (23%), stimulants and energy boosters (30%), and amino acid supplements (23%).

### 3.3. Intervention Effect on Dietary Supplement Use

Table 2 presents the binary logistic regression results for dietary supplement use in the experimental and control groups at baseline and post-intervention. Results indicated that at baseline, there were no significant differences in the odds of dietary supplement use between the experimental and control groups (OR = 3.4, 95% CI = 0.27 to 42.4, *p* = 0.34). At post-intervention, the experimental group showed a reduced likelihood of dietary supplement use compared to the control group (OR = 0.18, 95% CI = 0.01 to 1.76, *p* = 0.14), but this difference did not reach statistical significance. A post hoc power analysis for the post-intervention comparison indicated an achieved power of 61.4%. The odds ratio of 0.18 suggests a potentially meaningful reduction in supplement use in the experimental group. However, the limited sample size increases the likelihood of a Type II error, meaning that real effects may have gone undetected despite their practical importance.

Similarly, analysis for within-group differences between baseline and post-intervention did not yield any significant changes in the use of dietary supplements in the experimental group (OR = 0.12, 95% CI = 0.01 to 1.11, *p* = 0.061), as well as in the control group (OR = 2.2, 95% CI = 0.17 to 28.13, *p* = 0.54) (Table 3).

At the individual level, five participants in the experimental group transitioned from supplement users at baseline to non-users post-intervention, while 12 remained supplement users and one remained a non-user. In the control group, 10 participants remained supplement users, one transitioned from a non-user to a supplement user, and one remained a non-user.

## 4. Discussion

The objective of this study was to evaluate the effectiveness of an online educational intervention on dietary supplement use among varsity athletes. The results showed that no statistically significant effect of the intervention on dietary supplement use was demonstrated in this sample. While this intervention was successful in changing intentions and related determinants (attitudes and perceived behavioral control) towards dietary supplement use within the same population, seeing no change in behaviour is hardly surprising for a number of reasons [34].

Improving intentions towards or against a certain behaviour might not be enough to produce actual behavioral change. This is usually referred to as the intention–behaviour gap [36]. A very common example of the intention–behaviour gap is smoking cessation. While many smokers have the intentions to quit and are aware of the associated health risks, they often fail to or at least require multiple attempts to succeed. Icek Ajzen, the founder of the Theory of Planned Behaviour, states that a lack of “adequate” control over behaviour can sometimes make it impossible to perform an intended behaviour [26]. Kelly & Barker (2016) argue that human behaviours are far more complex than what can be altered or prevented through a simple introduction of new information. Rather, behaviours are shaped by an interplay of habit, automatic responses, convenience, cultural norms, and broader social environments [37]. Furthermore, the performance of an intended behaviour can be influenced by several factors, including lack of perseverance, limited resources, insufficient willpower, and the failure to obtain support from others [26].

It is well-documented in the literature that individuals often fail to act on their intentions, and that behaviours which persist are typically functional or convenient [37,38]. Sometimes, an intervention that is successful in changing intentions towards a certain behaviour may still fail to produce actual behaviour change due to control-related issues that inhibit the intended behaviour. In this case, a second different intervention is needed in which strategies that address control issues are incorporated [26]. For example, while varsity athletes may exhibit favourable intentions towards avoiding dietary supplement use, they might still rely on them during high-stress periods. In this study, the second questionnaire administration coincided with the University of Guelph’s final exams. Therefore, changing an established habit or adopting a food-first approach, which is typically more time-consuming, may not be functional enough during such periods. In this case, time constraints might override intentions and lead varsity athletes to choose a more convenient option. However, factors such as stress, time constraints, food availability, and convenience were not directly assessed in this study; therefore, their potential influence on dietary supplement use cannot be determined.

Another reason why a behavioral change was not induced by our intervention could be due to a limited change in intentions that was insufficient to cause a behavioral change. Intention is a direct predictor of behaviour; however, a large shift in intention is usually needed to cause a behaviour change [26,38]. Two meta-analyses have shown that even when interventions cause medium to large changes in intention, behaviour change is often only small to medium [39,40]. The relationship between changes in intentions and changes in dietary supplement use was not directly examined in the present study.

Another factor that may have contributed to the absence of behavioral change in our study relates to the participants’ stage of readiness for change. Stage-based models of behaviour change suggest that individuals move through distinct phases when attempting to adopt or eliminate a behaviour [41]. Perhaps the most popular stage model is the transtheoretical model, which suggests that there are six stages of change: precontemplation, contemplation, preparation, action, maintenance, and termination [42]. A distinct intervention is required at each stage to promote change and movement from one phase to another [26]. The Theory of Planned Behaviour does not disagree with the stage models perspective; however, it focuses on the motivational phase that leads to the formation of an intention [26]. This could also explain why improved intentions did not lead to a measurable change in dietary supplement use.

Control-enhancing techniques may therefore be critical in bridging this gap. For instance, action planning (e.g., specifying when and how to use food-first strategies before and after training sessions) can help athletes translate intentions into concrete behavior. Motivational interviewing, for example, can further strengthen commitment and resolve ambivalence, particularly in athletes who may be uncertain about abandoning supplement use. Similarly, habit formation tools, such as environmental prompts, cues, or substitution strategies (e.g., keeping healthy snacks available in training facilities), can cue automatic food-first behaviors and enhance long-term adherence.

It is generally easier to modify behaviours through the introduction of new beliefs rather than attacking existing ones. Additionally, the beliefs that influence behavior in real-life situations may differ from those that are accessible during hypothetical assessments of the Theory of Planned Behavior constructs [26,43]. Thus, in the case of an intention–behaviour gap, it is crucial to 1) address control issues that prevent acting on previously expressed intentions, and 2) prompt people to form an implementation intention. However, we must acknowledge that while we can assess certain aspects of control, we often lack sufficient information about all the relevant factors that might either facilitate or hinder the execution of the intended behavior.

Several alternative explanations should also be considered when interpreting the absence of a statistically significant intervention effect. The small sample size may have limited the statistical power to detect changes in dietary supplement use. In addition, assessing dietary supplement use as a broad binary outcome (user/non-user) may not have been sufficiently sensitive to capture changes in the types, frequency, or patterns of supplement use. The relatively short follow-up period may also have limited the opportunity for changes in intentions to translate into behavioural changes.

### Strengths & Limitations

This study has several strengths. It evaluated a theory-based online educational intervention designed specifically for varsity athletes and examined dietary supplement use as a behavioural outcome. The randomized study design and assessment of participants at baseline and post-intervention are additional strengths. Furthermore, the online delivery of the intervention provides a practical approach that may inform the development of future nutrition education interventions targeting athlete populations.

These results, however, should be considered in light of several limitations that may inform future research. First, the small sample size and unequal group sizes may have limited the statistical power of the analyses. Therefore, the findings should be interpreted as exploratory. The relatively short follow-up period may also have limited the ability to detect behavioural changes following the intervention. The analyses relied on self-reported data, which can introduce recall and reporting biases. However, this approach was necessary considering the nature of the study design and in order to ensure confidentiality. Additionally, the timing of the post-intervention questionnaire coincided with the final exams period at the University of Guelph. Behaviour change is usually hindered by obstacles that prevent people from carrying out their intentions, and time constraints are a significant barrier in this context. However, it was not possible to postpone data collection, because as time passes, an increasing number of intervening obstacles can change peoples’ beliefs. Additionally, the sample predominantly consisted of females, which may limit the generalizability of the findings. Finally, dietary supplement use was examined as a broad binary outcome, which did not account for differences in supplement type, purpose, frequency, dose, safety, or whether supplementation was medically or nutritionally indicated.

## 5. Conclusions

In this pilot study, no statistically significant effect of the four-week TPB-based online educational intervention on binary-defined dietary supplement use was demonstrated among varsity athletes. Given the small sample size, these findings should be interpreted as preliminary. Future studies with larger samples, informed by an a priori sample size calculation, and longer follow-up periods are needed to more reliably evaluate changes in dietary supplement use. Future research should also use more detailed measures of supplement use that consider supplement type, frequency, dose, indication, source of recommendation, and the use of certified versus non-certified products. Finally future studies may also benefit from directly assessing barriers to behavioural control and examining whether changes in TPB constructs mediate subsequent changes in behaviour.

## Figures and Tables

**Table 1:** Dietary supplement use prevalence at baseline for all participants (n = 30).

Category	Supplement	Use (%)
Vitamin/Mineral Supplements	Multivitamins	13%
	Calcium	10%
	Iron	23%
	Vitamin B12	20%
	Vitamin D	57%
	Vitamin C	53%
Protein Supplements	Protein bars/powders/shakes	43%
	Soy protein	13%
	Creatine	17%
	Casein protein	7%
	Whey protein	40%
Carbohydrate Supplements	Powders	20%
	Gels	33%
	Sports drinks	50%
Other Supplements	Fatty acid supplements	23%
	Stimulants/Energy boosters	30%
	Amino acid supplements	23%

**Table 2:** Comparison of dietary supplement use between the control and the experimental groups, at baseline and post-intervention using binary logistic regression.

Timepoint	Control (%) (95% CI)	Experimental (%) (95% CI)	Odds Ratio (95% CI)	*p*-Value
Baseline (n = 30)	83.3% (51–97%)	94.4% (74% to 100%)	3.4 (0.27 to 42.4)	*p* = 0.34
Post-intervention (n = 30)	91.7% (75–99%)	66.7% (41–87%)	0.18 (0.01 to 1.76)	*p* = 0.14

*p* < 0.05 considered statistically significant. CI: confidence interval. Logistic regression models were adjusted for age, gender, medical condition, level of education of parents or guardians, ethnic background, smoking habits, and alcohol consumption.

**Table 3:** Changes in dietary supplement use between baseline and post-intervention, within the control and the experimental groups using binary logistic regression.

Group	Baseline (%)	Post-Intervention (%)	Odds Ratio (95% CI)	*p*-Value
Experimental (n = 18)	94.4% (17/18)	66.7% (12/18)	0.12 (0.01 to 1.11)	*p* = 0.06
Control (n = 12)	83.3% (10/12)	91.7% (11/12)	2.2 (0.17 to 28.13)	*p* = 0.54

*p* < 0.05 considered statistically significant. CI: confidence interval. Logistic regression models were adjusted for age, gender, medical condition, level of education of parents or guardians, ethnic background, smoking habits, and alcohol consumption.

## Data Availability

The original contributions presented in the study are included in the article. The questionnaire is available upon request from the corresponding author.

## Abbreviations

The following abbreviations are used in this manuscript:

TPB	Theory of Planned Behaviour
WADA	World Anti-Doping Association
